# Physicians' information-seeking, appraising, and clinical decision-making practices for drug prescriptions: an exploratory study

**DOI:** 10.5195/jmla.2025.2082

**Published:** 2025-07-01

**Authors:** Akhi Nath, Julien Meyer, Mathieu Templier

**Affiliations:** 1 akhi.nath@torontomu.ca, Toronto Metropolitan University, Canada; 2 julien.meyer@torontomu.ca, Associate Professor at the School of Health Services Management, Toronto Metropolitan University, Canada; 3 mathieu.templier@fsa.ulaval.ca, Professor of Management Information Systems in the Faculty of Business Administration at Université Laval, Canada

**Keywords:** Evidence Based Practice, Information-Seeking, Clinical decision-making

## Abstract

**Objective:**

The purpose of this study is to understand the process of physicians' evidence-based clinical decision-making for new drug prescriptions.

**Methods:**

Eleven semi-structured interviews were conducted, and thematic coding was used for data analysis.

**Results:**

Several findings emerged. First, point-of-care information seeking focuses more on accessible and easy-to-use sources, such as medical websites, while out-of-practice searches encompass broader sources such as printed sources and extended networks. Medical websites are becoming preferred sources of information. Second, critical appraisal of information is performed passively by using pre-appraised information sources and referring to professional networks. Third, professional networks (i.e., specialists and senior colleagues) remain essential throughout the process and are pivotal for the decision to change prescription practices.

**Conclusions:**

Medical information systems that facilitate immediate access to summarized reliable evidence and feature real-time connectivity to the communities of practice can be an effective strategy for improving physicians' evidence-based practice for new drug prescriptions.

## INTRODUCTION

Evidence-based practice (EBP) requires physicians to be current with the best scientific knowledge produced by research. The National Academy of Medicine (NAM) identifies EBP as one of five core competencies for clinicians of all disciplines to ensure safe, patient-centered, timely, efficient, and equitable care [1]. However, the growing body of medical literature makes it difficult for physicians to stay current, contributing to outdated patient care. The ever-increasing accumulation of evidence and practitioners' inability to keep up to date is not new, especially in the medical field, where biomedical research information doubles every 20 years. For example, citation records in MEDLINE have risen to 981,270 in 2022 compared to just 579,041 in 2004 [2]. The exponential growth of information, coupled with rapid technological advancements and the increasing demand for interdisciplinary collaboration, is rapidly transforming the healthcare landscape, making it increasingly difficult for physicians to manage the overwhelming influx of information [3]. Despite physicians' positive attitudes toward evidence-based medicine (EBM), actual practice remains poor due to barriers such as lack of access to readily available applicable knowledge, lack of time to search for evidence, and lack of skills to identify, appraise, and clinically apply that knowledge [4-6].

The research-to-practice gap has been an ongoing issue, and despite continuous awareness and a push for evidence-based practice, it still exists [7]. Failure to use research evidence to inform decision-making is one of the reasons for the gap between best practices and physicians' actual prescribing practices [8], which manifests as overprescription [9] or underprescription of drugs [10-11]. Moreover, the gap between research evidence and practice not only deprives patients of receiving the best possible care but also leads to the waste of billions of dollars spent each year in healthcare research [5].

Translating evidence into practice requires the creation and dissemination of research information, as well as the active participation and effort of physicians in acquiring and applying the evidence both prior to and during decision-making [6]. In an environment like healthcare, where research evidence constantly accumulates from numerous sources, it remains unclear how physicians navigate these sources in the context of evolving technologies to inform their decision-making processes [12]. It is still not well understood how physicians choose information sources and seek information to find answers to their clinical questions, especially at the point-of-care (POC) [13].

To improve the uptake of research evidence by physicians at the point-of-care, it is essential first to understand how physicians, from their perspective, use information sources and other information technologies to inform their decisions. The purpose of this study is to provide deeper insights into the current practices of physicians in selecting, appraising, and applying information to inform drug treatment decisions, as well as the role of information technologies in these practices. We focus on drug-related information-seeking and application practices as drug therapy, or treatment-related clinical questions are more frequent for physicians in practice settings [3, 13].

For this purpose, the following research questions will be investigated:

- How do physicians choose information sources for their drug information needs?
- How do they determine the reliability and validity of the new information they find on drug prescriptions?
- How do they make the decision to apply the information in their practice?

## METHODOLOGY

### Theoretical Framework

**Figure 1:**
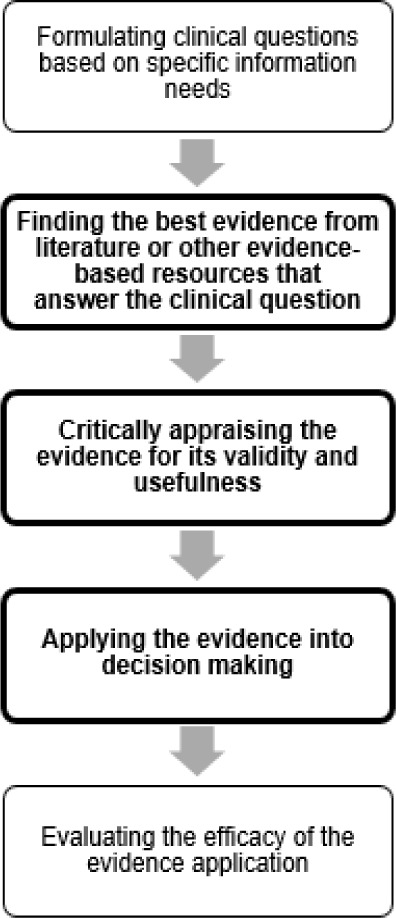
The Evidence-Based Medicine Model [15].

The concept of EBM was first introduced in 1992 in a research article by Guyatt et al [14]. EBM focuses on informing clinical decision-making based on the best available research evidence [15]. EBM has been defined as “the conscientious, explicit, and judicious use of current best evidence in making decisions about the care of individual patients” [16]. As our study focuses on evidence-based practices of individual practitioners, to investigate the physicians' process of acquiring and applying evidence-based information in practice, we have adapted the EBM model [15, 17]. The model consists of five essential steps, of which three steps of finding the best evidence (objective I), appraising the evidence (objective II), and applying the appraised evidence in clinical decision-making (objective III) will be explored in this study (figure 1).

### Study Design

A qualitative research design using semi-structured, open-ended interviews was employed to explore the context of drug prescription decisions. This approach is well-suited for health services research, allowing for an in-depth exploration of the research questions through physicians' narratives [18].

### Participant Recruitment

Participants were recruited using convenience sampling, targeting physicians with clinical practice experience prescribing drugs. To gather diverse perspectives, participants varied in age, gender, experience, practice areas, and qualifications. After obtaining Research Ethics Board approval from Toronto Metropolitan University (REB 2020-115), recruitment was conducted through social media (LinkedIn and Facebook), publicly available emails of physicians' practices, in-person visits to physicians' offices, and snowball sampling. Informed consent was obtained, and participants received a $20 Amazon gift card as compensation for their participation. The initial target was 20-30 participants, a number considered acceptable and recommended by experts in the qualitative research field [19-20]. Due to low response and limited resources, 11 were ultimately recruited, including family physicians, specialists, residents, and medical officers (“doctors who worked in hospitals and do not have a postgraduate qualification”) [21].

### Data Collection

An interview guide was developed based on the EBM model and previous similar studies [21] to include inquiries into the three particular themes: (i) how participants search for drug information, (ii) how they appraise and assess the information, and (iii) how it informs their decision-making and prescriptions. The interview questions were validated with expert reviews (e.g., trained qualitative researchers and a healthcare professional with knowledge of drug prescription practices). As the interviews progressed, the guide was updated with additional prompts, and a few questions were rephrased for clarity, keeping the main theme consistent, to ask respondents about their information-seeking behaviors in specific situations that emerged (see Appendix A).

Eleven participants were recruited, leading to eleven in-depth interviews between August 2021 and January 2023. One interview was conducted in person at the participant's workplace, and the other ones were conducted virtually through Zoom. Interviews lasted from 25 minutes to 1 hour, averaging 45 minutes. The interviews were recorded, leading to 447 minutes of audio recordings that were transcribed verbatim into 132 pages of transcripts using Microsoft Word Transcribe. Transcripts were reviewed for accuracy and anonymized by AN.

### Data Analysis

Data were analyzed using a thematic analysis approach. Researchers read the transcripts and notes taken during the interviews thoroughly to familiarize themselves with the data. Two researchers (AN and JM) independently coded the first 2 interviews in NVivo-12 and then discussed together to develop an initial list of codes. Subsequent interviews were coded using this list. If new themes emerged, those were added to the list after consulting with the research team. Then, the similar codes were collated into various emerging sub-themes, which were then placed under the three major themes established from the three major steps of the EBM model. Throughout the data analysis, codes, sub-themes, and themes were iteratively reviewed and discussed with the second researcher to resolve any discrepancies and disagreements.

## RESULTS

### Sample Description

Among the eleven respondents, seven were female, and four were male. Four of them were Family Physicians (FPs) (three independent practitioners and one resident), five were from specialized practice domains (one practicing geriatrician, two ophthalmologists, one otolaryngologist, and one psychiatry resident) and two were medical officers (internal medicine). Seven of the respondents are licensed to practice in Canada, and four are internationally trained physicians from Bangladesh. The practice years of the independent practitioners varied from 1 year to 18 years (table 1).

**Table 1:** Respondent Characteristics (n = 11)

Participant	Gender	Practice Domain and Country	Years of Experience	Practice Setting
FP-1	Male	Family Physician [Canada]	18 years	Group Practice Settings
SP-1	Male	Psychiatry [Canada]	First Year Residency	Academic Health Science Centre
FP-2	Female	Family Physician [Canada]	17 years	Group Practice Settings
SP-2	Female	Geriatrics [Canada]	3 years	Academic Health Science Centre
FP-3	Female	Family Physician [Canada]	Unspecified	University Community Medical Clinic
FP-4	Female	Family Physician [Canada]	Final Year Residency	Academic Family Health Team
SP-3	Female	Ophthalmologist [Canada]	1 year	Group Practice Settings
IMG-1	Female	Internal Medicine Medical Officer [Bangladesh]	4 years	University-affiliated Medical College and Hospital
IMG-2	Female	Internal Medicine Medical Officer [Bangladesh]	2 years	University-affiliated Medical College and Hospital
IMG-3	Male	Otolaryngologist [Bangladesh]	18 years	Solo Practice in a Private Clinic
IMG-4	Male	Ophthalmologist [Bangladesh]	6 years	Non-profitable Specialized Hospital

* FP, SP, and IMG refer to Family Physicians, Specialists, and International Medical Graduates respectively, and were used to identify the participants in the study.

The main themes are discussed below with some supportive quotes.

### Encountering a New Medication and Seeking Information

In our sample, participants mentioned that they were often introduced to new medications by visiting pharmaceutical sales representatives, at conferences, or, in a few instances, by patients.

Most of the time, participants did not actively seek information on it until they felt that this medication might satisfy their prescription needs. For instance, a patient might experience side effects with other medications and the physician was running out of alternatives. At that point, the physician would seek additional information about the drug, such as confirmation of its efficacy, side effects, dose, drug interactions, any available studies, legal approval, or insurance coverage.

To find answers to these clinical questions, physicians referred to different sources of drug information including paper-based and electronic resources such as medical websites, journals, MEDLINE/PubMed, product monographs, pharmaceutical handouts by pharmaceutical sales representatives as well as their professional networks such as colleagues, specialists, pharmacists. However, the selection of specific sources was highly contingent on the presence or absence of the patients in the room.

#### i) Information Seeking at Point-of-Care

At the point-of-care, during a patient encounter, when most clinical questions arise, physicians usually have limited time to look for information. They would then opt for fast and readily accessible sources such as peer-reviewed point-of-care subscription resources (eg., UpToDate, Dynamed), medical websites/apps (Medscape, Epocrates, Rx Files), product monographs, and pharmaceutical handouts.

If it's something I can quickly look up, I would just have the patient wait, check it, go back into my office, and then double-check the literature… certain websites are reliable, so we would just often quickly Google and go on those websites and then double-check that drug.

SP3

Some respondents even mentioned reaching out to interpersonal sources from their professional networks, such as colleagues, pharmacists, and specialists, given that they were immediately accessible at the point-of-care and perceived as reliable (table 2).

If I'm in the clinic and through our EMR system, I can actually send instant messages to him (Pharmacist) if he is working that day. So sometimes, you know, if I don't have time to go through the entire UpToDate or entire RX files, I often instant message him.

FP4

**Table 2:** Information Sources Used by The Participants

Categories	Information Sources	Used by	Physicians' Perceptions of the Sources	Used at
Electronic Resources	Web-based Medical Resources such as Prescription subscriptions (UpToDate, DynaMed), Medical Websites/Apps (Medscape, Rx Files)	FP1, SP1, SP2, FP3, FP4, SP3	Information availability, Time-consuming	Point-of-care
	MEDLINE/PubMed/University library websites	SP1, SP2, FP4	Accessibility	
Paper Resources	Product monographs, Pharmaceutical handouts	FP3, IMG3		
	Textbooks		Time-consuming, lack of applicability	
Interpersonal/Expert Knowledge Sources	Supervisors (for residents)	SP1	Reliable, Accessibility	Out of practice
	Specialists	SP1, FP4	Reliable, information availability	Point-of-care
	Practice-Based Small Groups/Opinion leaders	FP1, FP3, FP4	Reliable, accessibility	
	Colleagues	FP2	Accessibility	Out of practice
	Pharmacists	SP1, FP2, FP3	Accessibility, reliability	Point-of-care
Continuing Professional Development (CPD)	CME sessions	FP2, SP2, FP4	Reliable, information availability	Point-of-care, Out of practice
	Journals/professional associations newsletters	FP1, FP2	Reliability	Not specified
	Conferences	SP1, SP2, FP2, IMG4	Reliability, information availability	Point-of-care, Out of practice

#### ii) Information Seeking Outside the Practice Time

Point-of-care searches remain time-constrained, and physicians often conduct extended searches for information outside of their practice settings. This would include literature reviews on research databases or at their institutional libraries.

We generally refer to newer medications like articles that have been published, like randomized clinical trials, etc. So the primary literature, but again, that's really on our off time, we wouldn't really be doing this with the patient in the room, and if this is something that we're thinking, I would schedule a follow-up appointment. Then, I can do my own research before prescribing them.

SP1

During out-of-practice searches, they might also inquire into their professional networks such as colleagues, specialists, and pharmacists. For less pressing questions, they might get information by attending Continuing Medical Education sessions (CME), virtual lectures, presentations, pharma-sponsored dinners, seminars, academic and teaching sessions, scientific meetings, and conferences.

I would probably not prescribe that drug at all to the patient until I've done my thorough research or learned about it. It's really reading the articles…or reaching out to the drug reps… Or I could consult, like, maybe a senior colleague who used the drug before. Or I could do both these seminars that we have from time to time about educating us about different devices in ophthalmology, and different drugs in ophthalmology, and I would learn through that.

SP3

### Critically Appraising the Information

Participants emphasized ensuring the reliability and validity of any information related to a new drug. Only a few would appraise new evidence, while most respondents relied on sources that were already validated by other sources or experts, such as UpToDate, Medscape, and Dynamed.

Medscape is something that's easily accessible to us as physicians and that's something where we have evidence-based information about like side effects, dosing, frequency of drugs. So that's something we've used.

SP3

The perceived trustworthiness of the sources was more crucial in determining the reliability and validity of the information rather than the quality of the information itself. For instance, while respondents welcomed information from pharmaceutical sales representatives (PSR), it was not sufficient to alter their prescription practices. Before prescribing it, physicians will confirm the information with either colleagues or supervisors.

The drug reps no, I don't 100% believe everything basically. Their job is to sell the drug, right? So they're already biased. So I don't trust every drug rep. I take the information down, and I have to consult with another colleague or another clinician to really believe if a drug is actually effective because drug reps will; their job is to sell you the drug, so they will say everything positive about it. So I think my trust for drug reps isn't that great, but I think they are a good resource to bring new information on the table.

SP3

Engaging within their communities of practice provides an opportunity to discuss and validate any new drug or information. This could mean attending journal club sessions, conferences, or even dinners with colleagues.

One part of conferences or big meetings is abstract submission and research publications. Everyone will submit papers nationally and internationally, and you have a session whereby you will have a poster presentation or articles that are interested will be chosen for oral presentation. So there, people will present their newer research, and then you have the opportunity to ask questions or critically appraise your research.

SP2

### Applying the Information in Clinical Practice

Expert knowledge and opinions from professional networks (e.g., specialists, colleagues, supervisors, and pharmacists) highly influence prescription practices for new drugs.

The drug Rep would have dropped it off. I think I kind of looked at the box and then put it down, and I still, at that time, just did my regular. I kind of had my go-to sleep medications that I continued to use, and then I attended a virtual lecture…He was a psychiatrist, and he was sharing the details about this new medication and comparing it to the traditional ones. So actually after that discussion is when it started to become in my algorithm in my brain I guess of what I might use for sleep disorders.

FP3

Respondents, above all, valued the practical experience shared by their respected senior colleagues and specialists, which may sometimes even lead to certain biases about a particular drug:

One of the retina specialists was giving a talk through a seminar. He actually experimented with that drug in the States and, during that seminar, told us that the outcome of that drug is that it has many side effects, such that the benefits don't outweigh the risks. There were a few other ophthalmologists there at the dinner, and we all talked about that drug, and we kind of all agreed that. We all asked each other like, do you use it, do you use it? And all of us said nope, we haven't experimented with it. We've read about it. So after hearing this now, we're not going to use it until it's like last resort…so it is, I think, through the discussions, seminars, conferences, like a collaboration with our colleagues, that we learn. To some extent, we probably become biased toward using certain drugs over the other.

SP3

Sometimes, simple reassurance from experts can influence drug prescriptions by FPs or junior physicians:

I wasn't very sure about it, but when I recently prescribed it in psychiatry, a psychiatrist who was working with me last week. He has some experience, so he shared that he reassured me that this is a good medication I can actually prescribe.

FP4

Specialists were considered to have more clinical experience using a new drug in their domain and to have already evaluated all new evidence related to the new drug. It builds trust towards that new drug among other physicians, which influences the future use of that drug in practice:

Glaucoma specialists only see glaucoma patients. If there is a new glaucoma drug in the market, if I wanted to try that, I would first maybe read about the drug myself, and if I had questions, I would first seek advice from a glaucoma specialist to see how they tried it. If the glaucoma specialist says no, I don't trust this drug because of this and this reason then as a comprehensive ophthalmologist who sees some glaucoma patients…I would think, well, maybe the glaucoma specialist definitely has more experience than me. They are obviously more knowledgeable about this disease and the outcome, and they have reasons to believe why this drug is not effective. So then I would rely on their opinion, too, and if they don't use it, then I'd be very hesitant to use it without any kind of confirmation that it actually works.

SP3

## DISCUSSION

The results section detailed how physicians seek and evaluate information for drug prescriptions, both during practice and at POC, and provided insights on how physicians effectively find, appraise, and apply evidence to inform their drug prescribing decisions. Specifically, the following three key findings emerged:

Our first finding shows that at the point-of-care, physicians prefer immediately accessible sources such as medical websites or apps or proximal colleagues. In out-of-practice, they refer to primary literature, journal articles, or extended professional networks for detailed information. While reliability, easy accessibility, and convenience of use have been mentioned as some of the essential qualities of information sources by the participants, similar to other past studies [22-24], our finding suggests that easy accessibility is particularly critical at POC. Time constraints at point-of-care lead to an emphasis on accessible information sources, notably the immediate professional network and medical websites. These results reinforced how the timeliness of response is critical in determining which source to consult [25], depending on the practice setting.

Prior literature suggested conflicted findings as to physicians' preferences. Some argued that textbooks, journals, and colleagues' opinions were preferred [4, 22-23], while others suggested that web-based information sources were preferred over textbooks or journals [26-27]. Our results indicate that this may depend on the search timing, with medical websites/apps being preferred at point-of-care and journals and textbooks during out-of-practice searches because of time constraints. This seems likely considering that physicians spend only 2.2 mins searching for information during patient consultations instead of 32 minutes after consultation [28]. When an average patient consultation lasts less than 10 minutes, spending less time searching for information is reasonable [29]. Future research could confirm the hypothesis that at POC, easy accessibility to information is more highly correlated to satisfaction than during out-of-practice searches.

Our second finding is that physicians mostly rely on pre-appraised information sources or their professional networks to obtain reliable and valid information rather than appraising the evidence themselves. Most respondents relied on POC tools such as Dyna Med or UpToDate for evidence-based information. These websites are considered “pre-appraised” sources with already sorted higher-quality and up-to-date information [30]. Our study suggests a growing awareness and importance of such electronic sources compared to prior studies that reported low awareness and underutilization of such sources [4]. As these sources are effective at providing answers [31] and time-efficient for reliable information [32], this evolution is likely to continue.

Despite the pervasiveness of digital tools providing information on medications, when it comes to critically appraising medical information, our study suggests that physicians' professional network plays the most crucial role as they prefer to evaluate any new evidence through discussion within their communities of practice. This could mean consulting specialists, attending conferences, Continuing Medical Education events, journal club sessions, or other social events with their peers. This finding is in line with past research where specialists have been posited as a “shortcut” for general practitioners who lack the skills or time to assess all the evidence about drugs [33]. It also confirms a past study that found that physicians rely more on the authoritativeness of an information source to determine validity than on the content itself [34]. However, the literature provides limited support for physicians' critical appraisal practices, leaving an opportunity for future research in this area.

Our third finding is that the professional networks of physicians (i.e., specialists, senior colleagues, pharmacists) keep playing a decisive role in the final decision to prescribe a new medication. Physicians remain heavily influenced by the opinions of their professional networks, especially specialists or senior practitioners who were perceived to have more experience than them. The study suggests that the emergence of reliable and accessible online tools has not supplanted specialists yet. Validating their decisions with specialists could help physicians minimize their risks of applying new evidence (or a new drug) [35]. Physicians often require support, guidance, affirmation, and feedback beyond simply finding information that helps them make critical clinical decisions [22,36] and this behavior persists among technologically savvy physicians too. This raises the question of how reliable of a source senior practitioners are, especially when it comes to new medications. While this behavior protects patients from untrustworthy and unethical online information, it might also introduce cognitive and affective biases in their decision-making [37-38] and resistance to new drug adoption. As social norms evolve, future research could investigate the role of “shared decision-making” with patients, considering their opinions and preferences for any new drug.

Overall, our findings show that professional networks and, more generally, the reliance on peers for consequential decisions, such as new drug prescriptions, remain critical to enabling the translation of medical evidence into prescription practices.

As AI-driven decision tools emerge, that instantaneously synthesize large volumes of clinical evidence to provide physicians with relevant and up-to-date information based on individual patient characteristics [39], it remains to be seen whether and how these technologies will modify the current balance between fast POC practices and more time-consuming but more reliable out-of-practice information seeking practices.

Our study has implications for practice. The distinction between POC and out-of-practice information-seeking behaviors suggests a need for software providers to consider the dual needs of physicians: one modality of information searches focused on accessibility and simplicity and the other on comprehensiveness and reliability. This may involve integrating the two or considering bridges in the physicians' journey toward information seeking. Integrated options to consult experts in real time could also significantly encourage physicians to prescribe more state-of-the-art medications.

## LIMITATIONS

The study also has its limitations. First, the study had a small number of participants in specific locations and specialties and remains explorative. Future research should attempt to confirm its findings with a larger sample and possibly quantitative approaches. Such studies would investigate the role of other factors. For instance, residents, younger practitioners, and family physicians seemed more dependent on the opinions of their professional networks than specialists for new drug prescriptions. Second, interviewees' responses may have recall or response bias, reflecting socially desired practices rather than actual ones. To minimize bias, participants were asked to describe their most recent experiences. Observation of actual drug-prescription practices in future studies would alleviate that limitation. Finally, the data were collected during the pandemic over the course of 18 months, which may have altered respondents' practices and views towards more virtual information-seeking behaviors.

## CONCLUSION

This study has provided preliminary but valuable insights into physicians' drug-related information-seeking and clinical decision-making practices. By taking the physicians' perspective, we captured and documented a range of POC and out-of-practice evidence-seeking, appraisal, and use practices. As electronic and web-based resources continue to evolve and become more pervasive at the POC, it may be tempting to assume they will significantly influence physicians' decision-making for drug prescriptions at POC. Studies like this remind us that context and processes are critical in determining what information sources will be selected and whether they will be acted upon or dismissed. This will become even more important as the promises of these resources, coupled with the emerging disruption by artificial intelligence-based tools, expand if we want to ensure that the gap between evidence-based medicine and actual practice does not widen.

## Data Availability

Data associated with this article are available to researchers at https://borealisdata.ca/dataset.xhtml?persistentId=doi:10.5683/SP3/GG45SJ&version=DRAFT. Access to the data can be requested from the corresponding authors.
