# Supplementary material for: Physicians' information-seeking, appraising, and clinical decision-making practices for drug prescriptions: an exploratory study
Source: J Med Libr Assoc. 2025 Aug 1;113(3):224–32. doi: 10.5195/jmla.2025.2082 (PMC12369961; doi:10.5195/jmla.2025.2082)
Supplement: Supplementary file 1 — Appendix A [file jmla-113-3-224-s01.docx]

**Appendix A**

**Interview Guide**

**Introduction**

Hi, Thank you for taking the time to talk to me today. My name is ….. I am a graduate student at
[redacted] University working on a project named “Evaluation of an information system to transfer
research knowledge on medications to physicians” with researchers from [redacted] University and
[redacted] University partnering with [redacted], a startup company designing solutions for easy access
to drug information for physicians. The research is funded by the [redacted] and has been reviewed and approved by the Research Ethics Boards of [redacted] University [redacted] and [redacted] University [redacted].

The purpose of this qualitative interview is to understand how doctors acquire new knowledge of
medications and apply it in their practices. Real-life examples will help us to understand the process, you don't have to describe any patient specifics, we are more interested in the process you go through from arising a question to finding information to practice it in real practice settings.

Before we start, I want to let you know that if you want to stop the interview anytime or you
want to take a break, you can and if I was to ask you a question you don’t want to answer, please
feel free to tell me that; it’s absolutely fine. Also, for the research purpose, our conversation will
be audio-recorded and I assure you that only the research team will have access to this record. I
would like to make it clear that our study is independent of any influence from any company or
pharmaceutical. The data will be published anonymously so that the identity of the speaker
would not be published. Do you agree to proceed with the interview under these conditions?
Do you have any questions for me before we start?

**Interview Questions:**

Interview Questions:

- Can you please briefly tell me about your current position (Practice Domain/years of

practice/Practice Setting)?

- Could you please tell me about a patient case you recently managed where you have prescribed a new medication or a new medication in a different context for the first time?

If yes-walk me through the process from getting to learn about the medication to prescribing it to the patient.

**Possible prompts**-

- When and How did you get to learn about the medication?
- How did you decide to practice it/ignore it (learn more about the medication)?

**Information Seeking:**

- Where did you find that information?
- Why did you search here, not other sources (how physicians weighed different sources of information)?
- When and where did you conduct the search - (during patient consultation or afterward)
- How long did it take you to find an answer to that clinical question?
- How would you describe your experience in terms of fulfilling your information need?
- Is there a case where you did not find a satisfying answer? (If you did not find a clinical answer), what did you do?
- Does it always work like that? Is there any instance where you do the search differently?

**Critical Appraisal:**

- How did you know the info is valid, reliable, and applicable to your patient? Why did you decide to follow or not follow the evidence/information?
- Does it differ for different sources (only compare the sources they mentioned, e.g., electronic vs colleagues)
- Can you recall a time when different sources gave conflicting advice? How did you resolve that?

**Clinical decision-making:**

- How did you decide to use it for your specific patient case? (What/who influenced your decision?) or,

(How did you process the info to apply to your patient (patient conditions, customize the drug dose, patient preference)

**Additional Information**

- Can you think of another instance when you prescribed or discontinued a new medication or changed your prescription practice based on new information?
- What do you think could have improved your information-seeking and practicing experience at any stage of this process?

*If the respondent did not prescribe a new medication recently-

Could you please tell me about a patient case you recently managed where you have looked for new evidence/information related to drugs (e.g. Medications to COVID-19 patients) and decided your treatment plan based on that evidence? What kind of information did you look for? How did you address those? Repeat Step 3 questions.

- Is there anything else you would like to add?

**Demographic Questions**-

Country/Region of practice

Specialty area

Years of practice experience

Practice settings
